# Assessing the Impact of Primary Care-Led Optimisation Clinics in the Management of Type 2 Diabetes: A Service Redesign Pilot Study

**DOI:** 10.7759/cureus.47365

**Published:** 2023-10-20

**Authors:** Orestes Couppis, Faiz Juneja, Naheed Hussain, Zoe Davies

**Affiliations:** 1 General Practice, Leyton Healthcare, London, GBR; 2 Pharmacy, Leyton Healthcare, London, GBR; 3 Diabetes and Endocrinology, Whipps Cross Hospital, London, GBR

**Keywords:** behaviour, primary care network, pcn, general practice, optimisation, primary care, gp, type 2, clinics, diabetes

## Abstract

Background

Many individuals diagnosed with type 2 diabetes (T2D) in the London Borough of Waltham Forest, treated solely with oral hypoglycaemic medications (OHAs), exhibit increased levels of glycated haemoglobin (HbA1c). While specialised community and secondary care clinics are at full capacity, a gap exists for dedicated diabetes optimisation services at the primary care level. This study aimed to launch a remote Primary Care Network (PCN)-based clinic during the coronavirus disease 2019 (COVID-19) pandemic to enhance the management of OHAs, introduce motivational interviewing, and incorporate patient empowerment strategies in tandem with a secondary care endocrinologist. The primary objective was to evaluate the impact on HbA1c levels and other metabolic parameters. Concurrently, the “behaviour change model” served to measure patient engagement.

Methodology

We recruited 43 patients in this study, each undergoing a 30-minute consultation focused on diabetes management. A dedicated administrator ensured patient engagement and a three-month follow-up with repeat metabolic profile testing. Sustained, high-quality care was upheld through bimonthly remote consultations, receiving expertise from an endocrinology consultant.

Results

Of the pilot’s 38 patients managed solely with OHAs, 31 achieved an HbA1c reduction of more than 11 mmol/mol. The overall median reduction for the entire cohort was significant (initially 88 mmol/mol versus 70 mmol/mol, p < 0.0001). Triglyceride levels also saw a notable median decline (1.56 mmol/L down to 1.20 mmol/L, p = 0.0247). Of the 38 completing the pilot, 14 had behavioural stages recorded both initially and at follow-up. Employing motivational interviewing led to significant diabetes-related behavioural changes in 11 of the 14 patients.

Conclusions

A PCN-based optimisation clinic, augmented with active recall strategies, was a cost-effective method to boost awareness, self-management, and glycaemic control among individuals with T2D. These PCN-led clinics orchestrated by primary care clinicians offer a streamlined solution for achieving treatment benchmarks even amid the challenges of the COVID-19 pandemic.

## Introduction

Type 2 diabetes (T2D) is a chronic metabolic disorder marked by insulin resistance and impaired glucose regulation, placing a significant and escalating strain on the National Health Service (NHS) of the United Kingdom [1]. Factors such as genetics, lifestyle choices, and obesity influence the progression of T2D. With the rising prevalence of T2D, implications arise for both patient care and the distribution of healthcare resources. Given the central role of the NHS in diagnosing, managing, and preventing T2D, it is imperative to grasp the intricacies of this condition within the United Kingdom’s healthcare framework.

The London Borough of Waltham Forest experienced 852 referrals during 2018-2019 (unpublished data: Clinical Effectiveness Group. 2018-2019 Referral Data; 2019). This number encompasses referrals from all eight Primary Care Networks (PCNs) within the borough. Of these, the PCN in this study accounted for 157 secondary care referrals, intensifying the demands on an already stretched secondary care system. The community diabetes team, managed by the North East London Foundation Trust, has been in operation for several years. Like other healthcare services, it possesses limited capacity and faces challenges with managing the increased volume of referrals.

Current secondary care referral criteria emphasise the “super six criteria,” which include diabetic patients with stage 4 chronic renal disease, those with gestational or pre-pregnancy diabetes, patients with type 1 diabetes, patients undergoing insulin pump therapy, individuals with severe foot disease, and those suspected of having genetic or autoimmune diabetes [2]. With the continued increase in T2D prevalence, addressing the needs of patients outside these criteria necessitates greater resources and expertise at the primary care or community level.

This pilot study aimed to evaluate the efficacy of a dedicated diabetes optimisation consultation within primary care, concentrating on reducing glycated haemoglobin (HbA1c) levels and related metabolic indicators associated with diabetes. The study allocated resources to hire a primary care physician and a dedicated administrator over a nine-month period. The study’s primary goal was to engage patients, streamline the recall process, and offer extended consultations for individuals whose HbA1c levels surpassed 75 mmol/mol and who were not undergoing injectable therapy. Incorporating bimonthly virtual endocrinologist reviews aimed to deliver specialised advice promptly, elevating care quality and promoting ongoing learning. Ultimately, the intention was to create effective management strategies and enhance HbA1c and metabolic outcomes, thereby decreasing secondary care referrals and fostering behaviour beneficial for diabetes management. We also aimed to highlight the role of a dedicated administrator in bolstering patient engagement and clinic efficiency. The goal of using motivational interviews and patient empowerment techniques during thorough consultations was to fortify the chances of sustained behaviour change and curtail complications tied to T2D. This research was presented as an abstract in Diabetes Medicine Volume 40 Abstracts of the Diabetes UK Professional Conference 2023, Exhibition Centre Liverpool in April 2023.

## Materials and methods

After consultation with the Chair and the Clinical Lead of the Clinical Commissioning Group within the NHS and a thorough consideration of ethical implications, it was concluded that formal ethical approval was not necessary, given the nature of this project as a service redesign pilot study.

Throughout the study, patients received consistent management following established clinical protocols that conformed to national guidelines for diabetes care, maintaining adherence to accepted standards without any deviations in patient care.

The PCN, serving 35,000 patients, received £10,000 in funding for the optimisation pilot. This grant facilitated the hiring of a primary care physician and an administrator. Furthermore, a secondary care endocrinology consultant voluntarily provided their expertise to the pilot. The pilot, which ran successfully for nine months, comprised 15 optimisation clinics, four virtual consultant clinics, and six PCN meetings, collaboratively ensuring efficient study implementation. Optimisation clinics were locally devised, embodying patients from every practice within the PCN. A dedicated PCN administrator spearheaded the proactive recall of hard-to-reach patients. Participating individuals each underwent an initial 30-minute consultation tailored to the comprehensive needs of diabetic patients.

Every two months, a consultant endocrinologist from Whipps Cross Hospital (London, UK) supplied expert management and guidance. While this expertise is expected to bolster diabetes management, the focus shifted towards extended diabetes-centric consultations steered by primary care providers, where motivational interviewing and patient empowerment techniques could be employed effectively [3-5].

Due to the coronavirus disease 2019 (COVID-19) pandemic, the optimisation clinics were held via telephone throughout this pilot. These were four-hour sessions conducted biweekly at a PCN practice. Initial plans favoured in-person over telephone consultations. Systems such as EMIS and SystmOne granted secure access to electronic medical records for each practice. Striving for equitable representation from every practice, extended consultations were provided to the identified cohort.

The Clinical Effectiveness Group (CEG) set patient selection criteria, enabling practices to pinpoint specific patient groups. The securely password-protected database enabled the optimisation clinic physician to access this cohort. The pilot’s goal was to engage around 60 patients. Bi-monthly two-hour sessions with endocrinologists allowed discussions on individual patient care, ensuring the cohort’s access to secondary care consultants, which was vital for the pilot’s success.

In this study, we set a predefined target sample size of 60 patients based on the available funding. Our recruitment efforts resulted in the enrolment of 43 patients, and, of this cohort, 38 patients completed the study. The largest proportion of participants self-identified as South Asian, comprising 69.7% of the total, followed by those of Caucasian ethnicity at 18.6%, Afro-Caribbean at 9.3%, and individuals from other ethnic backgrounds at 2.4%. The average age of our study participants was 57 years, with age ranging from 37 to 81 years. Gender distribution among the participants was approximately equal, with 53.5% male and 46.5% female. The participants showcased diverse comorbidities and socioeconomic backgrounds, offering a well-rounded representation of the local area.

The study’s exclusion criteria encompassed individuals who had an HbA1c level below 75 mmol/mol within the preceding three months, those receiving injectable therapy, and those who were not actively participating in the pilot study.

Patient data were safeguarded via password protection and relayed only through secure NHS mail servers. The administrator was instrumental in managing numerous tasks, such as scheduling patient meetings, updating the database, and ensuring patients underwent timely tests, guaranteeing optimal clinical contact.

Follow-up clinics took place three to four months after the initial consultation. During these sessions, patients were given 15-minute telephone appointments and subsequent HbA1c and lipid profile tests. Where feasible, data including body mass index (BMI), blood pressure, and the patient’s stage in the “behaviour change model” were documented, allowing ongoing care, regular medication reviews, and opportunities to reinforce beneficial behaviours.

Patients in the statistical analysis attended the initial consultation, underwent metabolic testing, and participated in follow-ups. For long-term management, patients returned to their primary care practices, which committed to reviewing them within six months post-pilot, including renewed blood tests to monitor metabolic trends.

The CEG curated a cohort of T2D patients registered within the PCN, not on injectable therapy, and with HbA1c levels above 75 mmol/mol, measured six months before the pilot’s commencement. Those with measurements within the last three months were prioritised. If a quota of 15 patients from a practice was not met, those with HbA1c measurements three to six months before the pilot were considered.

The PCN’s administrator sent potential participants an invitation letter detailing the pilot study, which commenced in January 2021. The constantly updated database encompassed appointment dates, HbA1c levels, and other relevant metrics.

Post-consultation, vital information from each session was logged for analysis. Patients received key details through text messages, including sick-day guidelines and dietary advice. A designated email ensured continuity of care under the optimisation clinic physician’s oversight.

After their last optimisation clinic visit, patients were scheduled for a follow-up after a minimum of three months. Necessary tests were repeated before these appointments upon the PCN administrator’s initiation. If the patients needed referrals to secondary care or dietary specialists, the clinic doctor and administrator managed the process. The database was promptly updated. Once the pilot concluded or if patients disengaged, they returned to their native primary care practices for ongoing care.

## Results

Table 1 displays preliminary search outcomes. Patients with HbA1c levels below 75 mmol/mol in the last three months, those on injectable therapy, or those not actively participating were excluded from the pilot.

**Table 1:** Number of patients registered, diagnosed with diabetes, and with a recent HbA1c blood test across three practices. T2D: type 2 diabetes; HbA1c: glycated haemoglobin

Location	Currently registered (n)	T2D diagnosed over 12 months ago (n)	Excluding injectable medication (n)	HbA1c >75 mmol/mol in the past		
				3 months, n (%)	6 months, n (%)	12 months, n (%)
Practice 1	15271	785	623	28 (4.49%)	39 (6.26%)	53 (8.51%)
Practice 2	11915	737	616	56 (9.10%)	78 (12.70%)	96 (15.60%)
Practice 3	9780	645	549	10 (1.80%)	28 (5.10%)	42 (7.70%)
Total	36966	2167	1788	94 (5.30%)	145 (8.10%)	191 (10.70%)

In the past three months, we identified 94 patients with HbA1c levels above 75 mmol/mol who were not on injectable therapy. Though we aimed to recruit 60 patients, only 43 consented and completed the enrolment process. Of these, 38 underwent an extended initial consultation and a follow-up assessment, contributing data to the pilot. Five participants, however, did not complete the follow-up.

**Figure 1:**
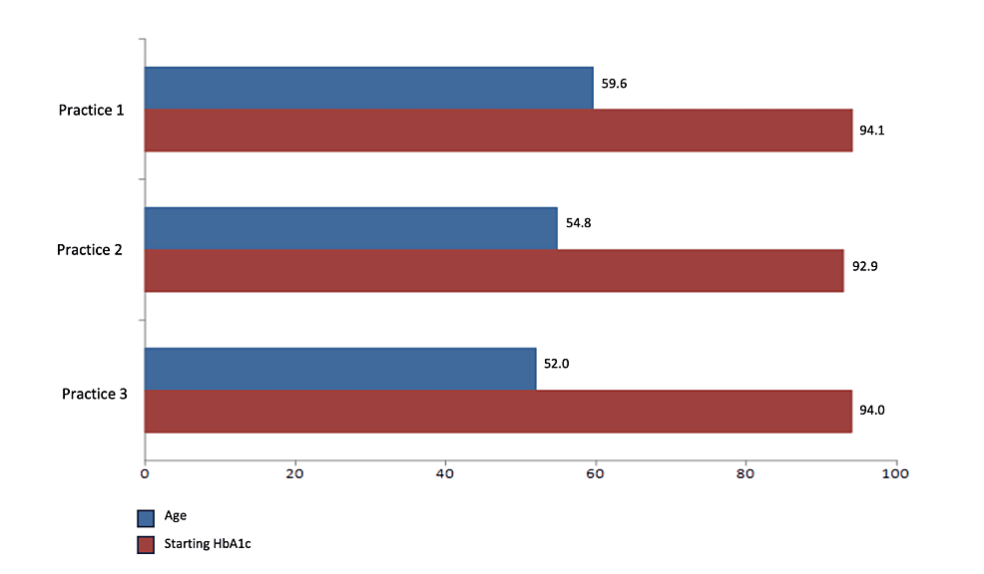
Comparison of age and initial pre-pilot HbA1c across three practices. HbA1c: glycated haemoglobin

The average age of patients in Practice 1, Practice 2, and Practice 3 was 59.6, 54.8, and 52.0 years, respectively. The average initial HbA1c levels across the three practices were 94.1, 92.9, and 94.0 mmol/mol, respectively (Figure 1).

**Figure 2:**
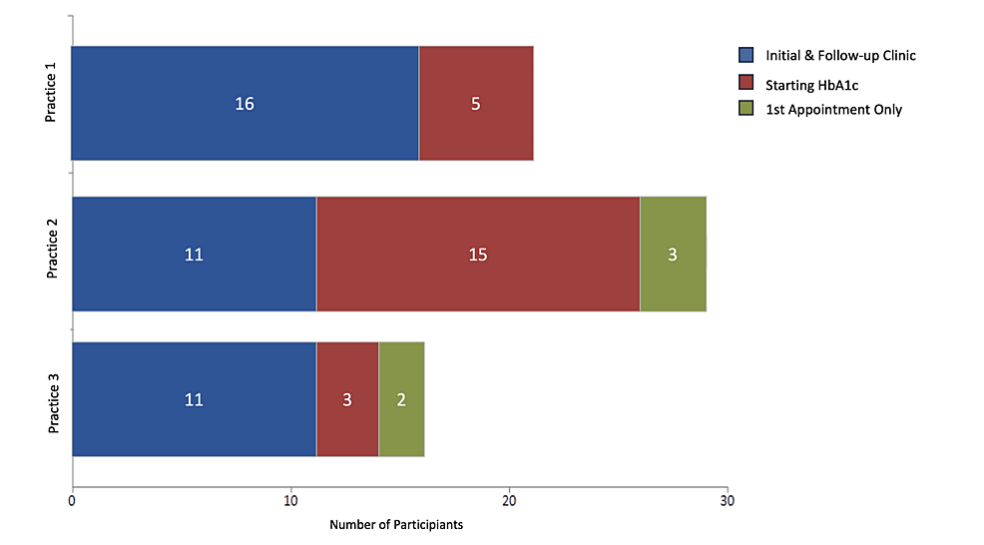
Comparison of the engagement with the pilot study across three practices. HbA1c: glycated haemoglobin

Regarding engagement, 16 patients participated from Practice 1, 11 from Practice 2, and 11 from Practice 3. Practice 2 saw three patients drop out after their initial consultation, while Practice 3 lost two (Figure 2). Patients with an initial HbA1c value who did not meet the participation criteria were excluded from the final dataset of the pilot study. Among those who initially engaged but later dropped out, reasons included relocating abroad or changing residences.

**Figure 3:**
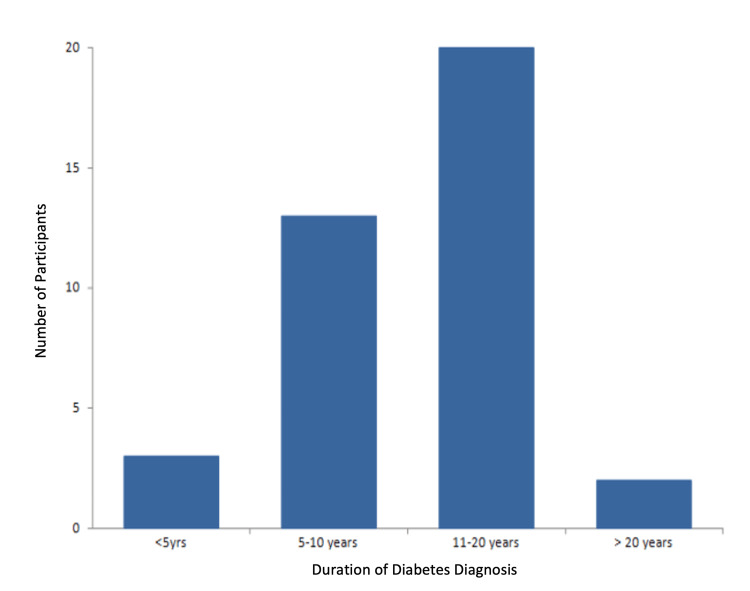
Duration of diabetes diagnoses among study participants.

Of the 38 patients, 20 had been diagnosed with diabetes between 11 and 20 years prior. Twelve had a five to 10-year history, three had been diagnosed within the last five years, and two had been living with diabetes for over 20 years (Figure 3).

**Figure 4:**
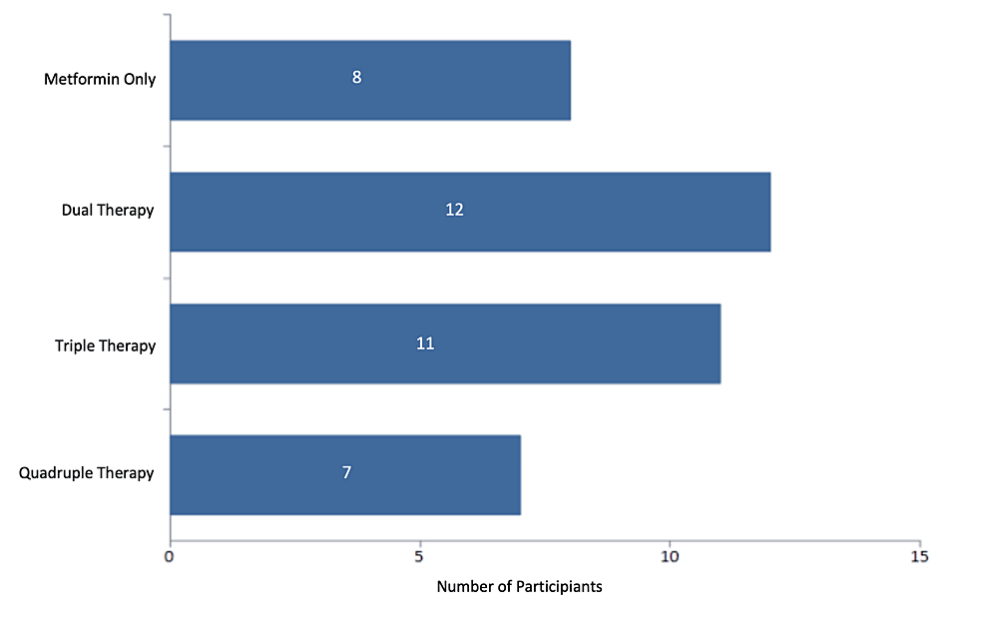
Oral hypoglycaemic burden.

Eight patients were solely on metformin, while seven took a combination of metformin, dipeptidyl peptidase 4 inhibitor (DPP4), sodium-glucose co-transporter-2 inhibitor (SGLT2), and sulphonylurea. The remaining 23 patients took two or three oral hypoglycaemic agents (OHAs) (Figure 4).

**Figure 5:**
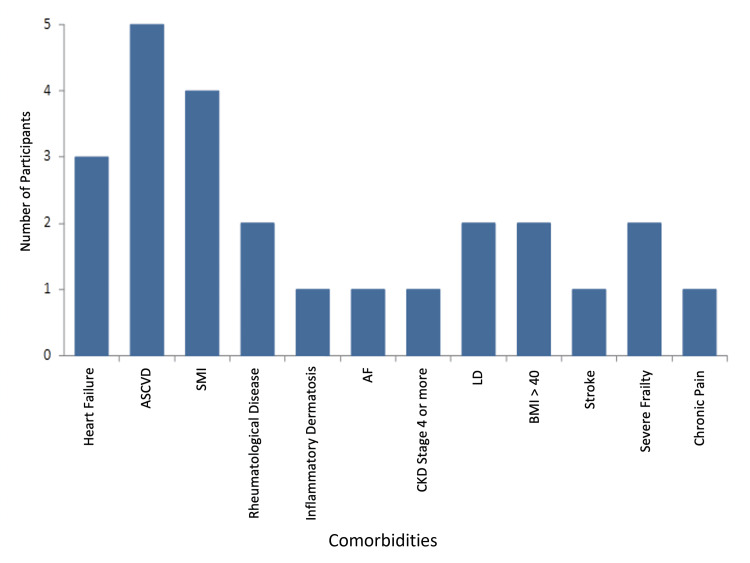
Comorbidities of the cohort (excluding hypertension, obesity, and dyslipidaemia). ASCVD: atherosclerotic cardiovascular disease; SMI: serious mental illness; AF: atrial fibrillation; CKD: chronic kidney disease; LD: learning difficulty; BMI: body mass index

Hypertension, dyslipidaemia, and high BMI were common in this cohort. Learning difficulties and serious mental illness were present in 16% of the patients, followed by atherosclerotic cardiovascular disease (13%) and heart failure (9%) (Figure 5).

Effects of intervention on metabolic parameters

**Figure 6:**
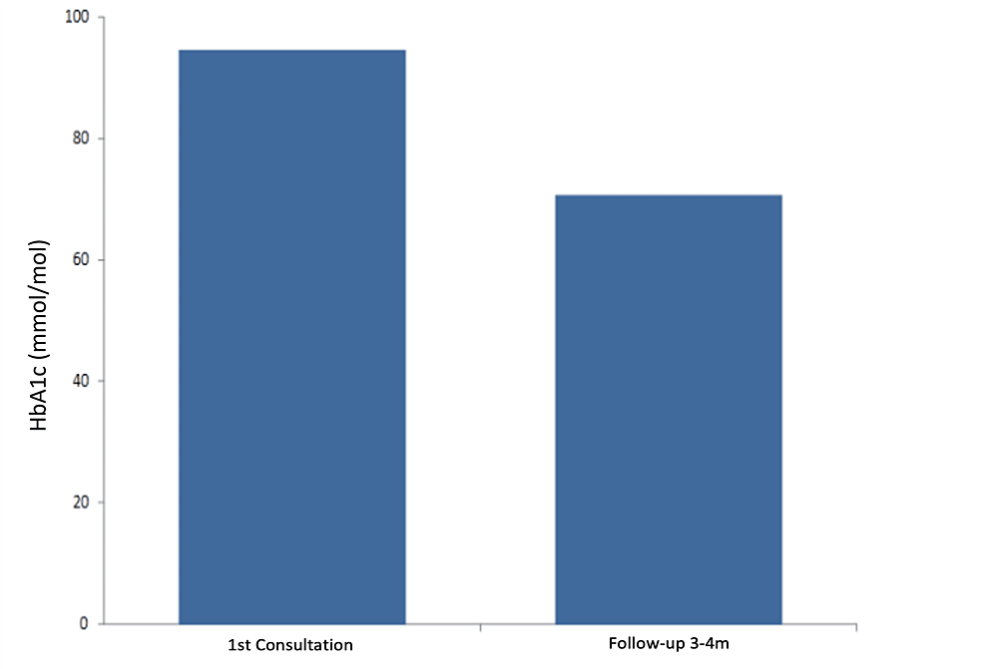
Pre- and post-optimisation pilot mean HbA1c results. HbA1c: glycated haemoglobin

**Figure 7:**
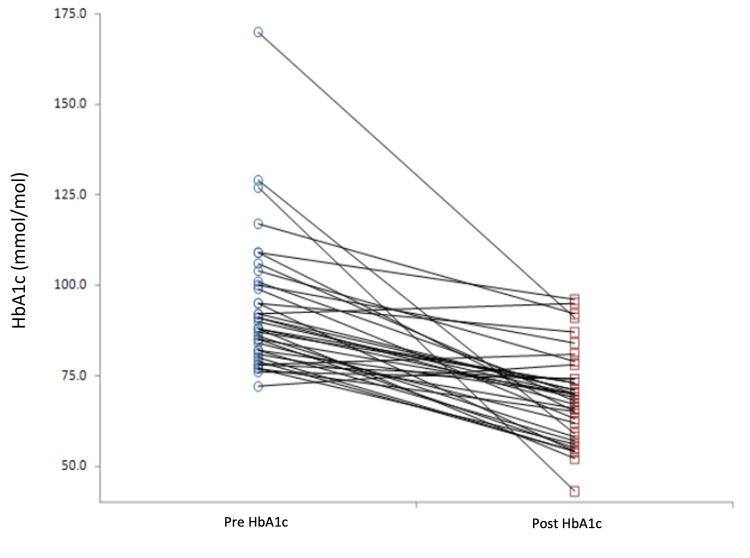
Ladder plot of pre- and post-optimisation pilot HbA1c results. HbA1c: glycated haemoglobin

**Figure 8:**
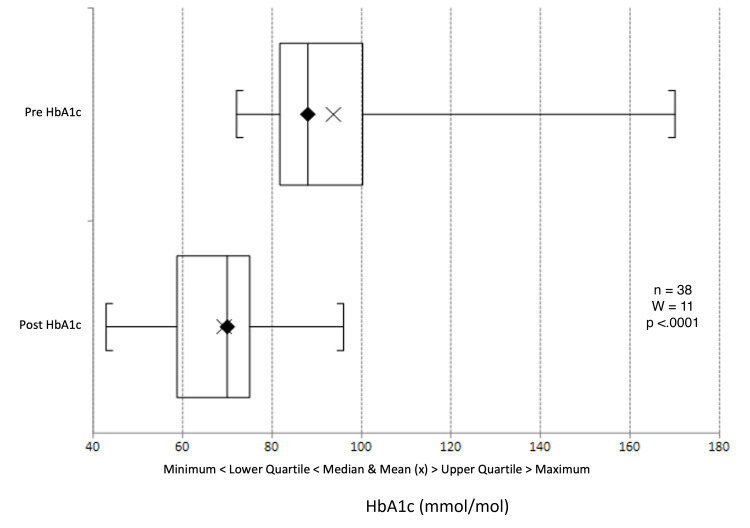
Box plot representation and Wilcoxon signed-rank test of pre- and post-intervention HbA1c results. HbA1c: glycated haemoglobin

We observed a statistically significant median decrease in HbA1c levels from 88 to 70 mmol/mol (average 93.8 to 69.4, p < 0.0001) (Figures 6-8). The three practices showed an HbA1c reduction of 22 mmol/mol, 21 mmol/mol, and 21 mmol/mol, respectively (Table 2). Notably, 31 of the 38 patients experienced an HbA1c reduction exceeding 11 mmol/mol.

**Table 2:** Pre- and post-optimisation changes in HbA1c values across three practices. ^a^: 95% CI: 0.043958 to 0.302806; mean difference: 22 (CI: 12 to 31). ^b^: 95% CI: 0.059019 to 0.406828; mean difference: 21 (CI: 6 to 35). ^c^: 95% CI: 0.017142 to 0.295826; mean difference: 21 (CI: 15 to 36). HbA1c: glycated haemoglobin; CI: confidence interval; NR: not recorded

Data collection event	Practice 1			Practice 2			Practice 3		
	Pre-HbA1c (mmol/mol)	Post-HbA1c (mmol/mol)	P-value	Pre-HbA1c (mmol/mol)	Post-HbA1c (mmol/mol)	P-evalue	Pre-HbA1c (mmol/mol)	Post-HbA1c (mmol/mol)	P-value
1	109	65	<0.0001^a^	127	43	0.0062^b^	120	116	0.0002^c^
2	90	70		72	78		95	54	
3	80	54		117	92		91	71	
4	85	55		86	58		85	70	
5	109	96		101	70		129	59	
6	84	62		100	84		82	70	
7	170	91		95	87		91	67	
8	106	68		92	95		88	69	
9	77	65		99	63		92	71	
10	88	73		81	66		78	74	
11	104	79		76	74		88	52	
12	87	73		NR	NR		NR	NR	
13	79	54		NR	NR		NR	NR	
14	82	57		NR	NR		NR	NR	
15	77	56		NR	NR		NR	NR	
16	78	81		NR	NR		NR	NR	

**Figure 9:**
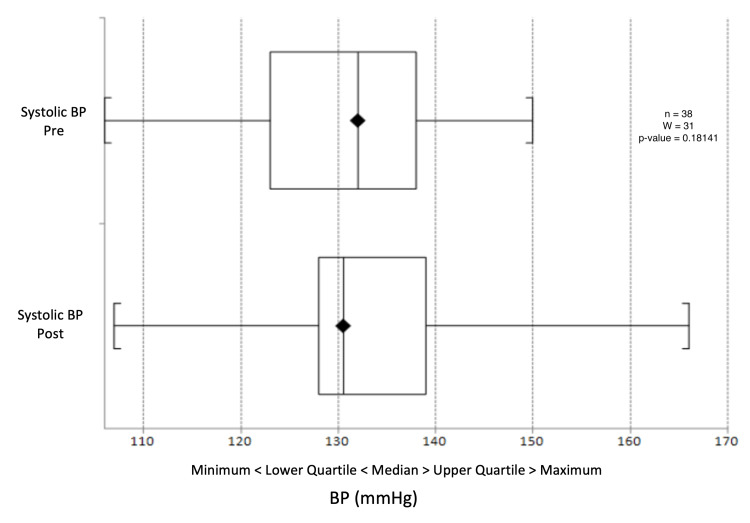
Box plot representation and Wilcoxon signed-rank test of pre- and post-intervention systolic BP results. BP: blood pressure

**Figure 10:**
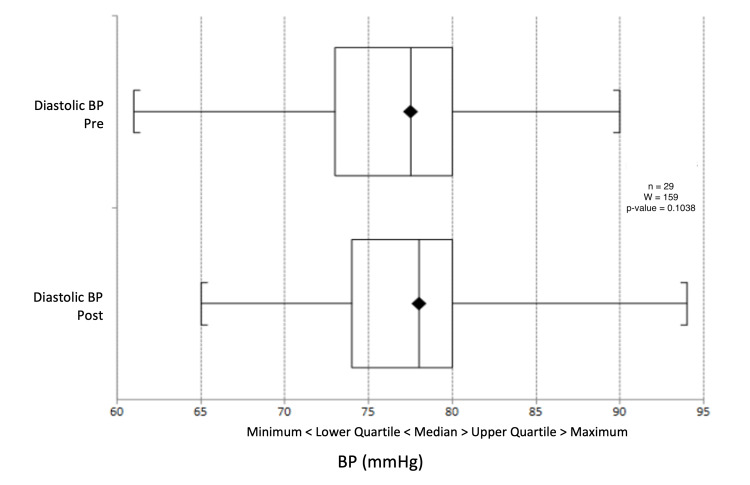
Box plot representation and Wilcoxon signed-rank test of pre- and post-intervention diastolic BP results. BP: blood pressure

**Figure 11:**
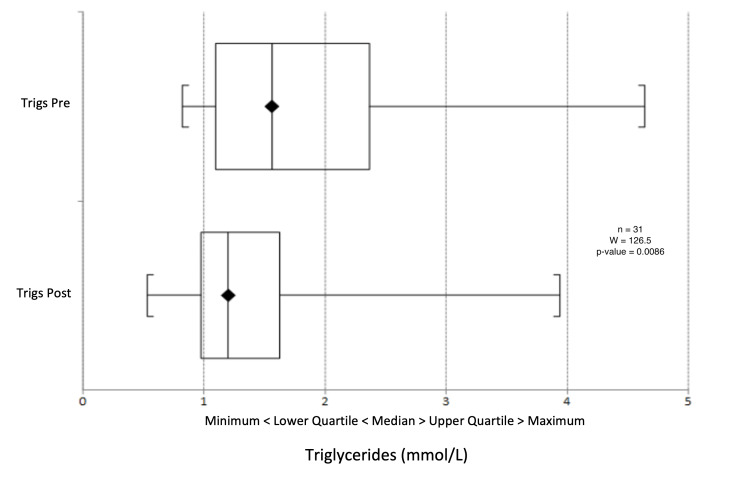
Box plot representation and Wilcoxon signed-rank test of pre- and post-intervention triglyceride results.

**Figure 12:**
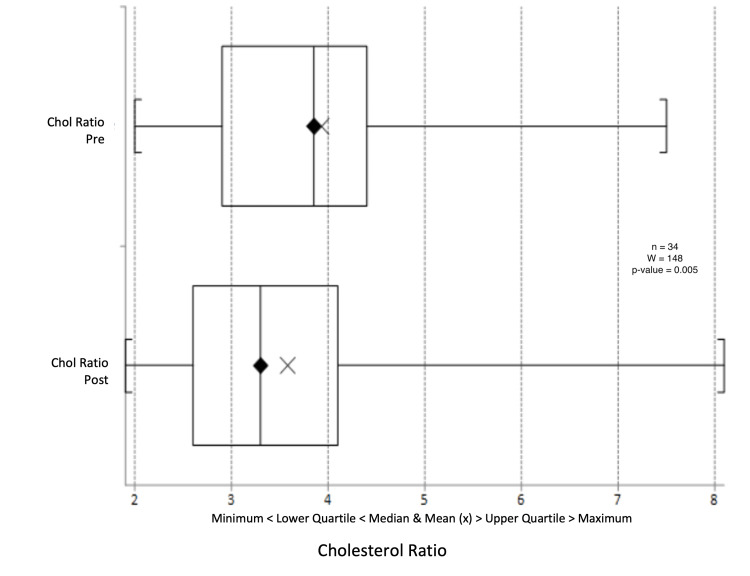
Box plot representation and Wilcoxon signed-rank test of pre- and post-intervention cholesterol ratio results.

**Figure 13:**
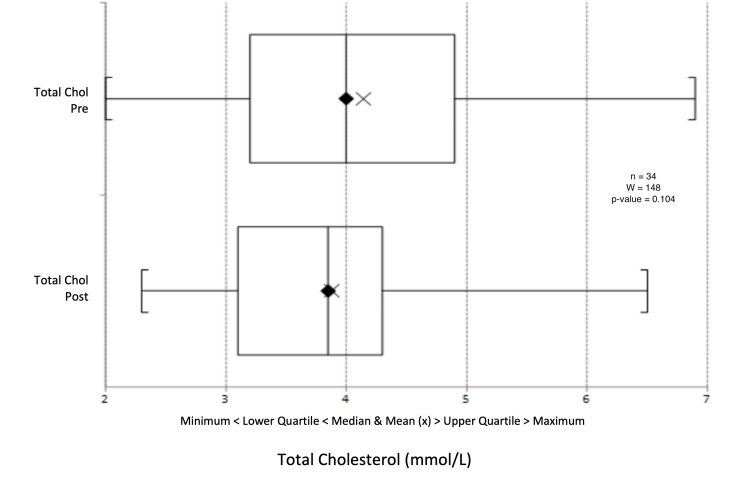
Box plot representation and Wilcoxon signed-rank test of pre- and post-intervention total cholesterol results.

**Figure 14:**
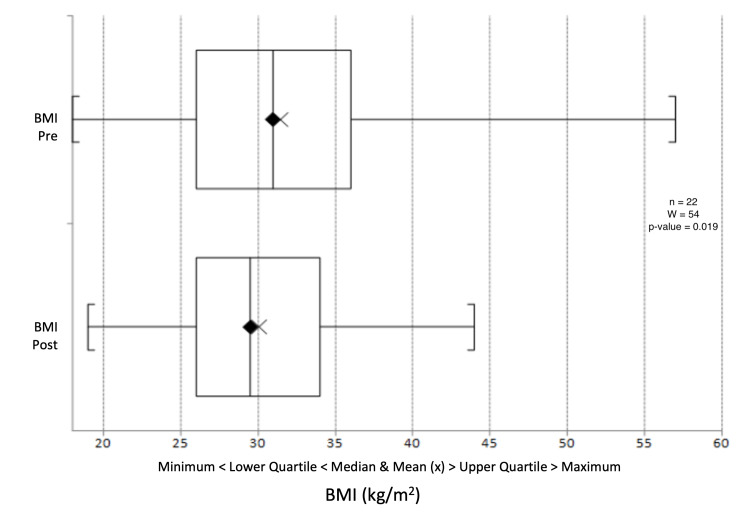
Box plot representation and Wilcoxon signed-rank test of pre- and post-intervention BMI results. BMI: body mass index

There was a median reduction in systolic blood pressure from 132 mmHg to 130.5 mmHg (p = 0.603) (Figure 9) and a change in diastolic blood pressure from 77.5 mmHg to 78 mmHg (p = 0.391) (Figure 10). Triglyceride levels decreased significantly from 1.56 to 1.20 mmol/L (p = 0.0247) (Figure 11). Cholesterol levels dropped from 3.38 to 3.30 mmol/L (p = 0.207) (Figure 12), and total cholesterol levels decreased from 4.00 to 3.85 mmol/L (p = 0.415) (Figure 13). The median BMI reduced from 30.95 kg/m^2^ to 29.5 kg/m^2^ (p = 0.533), although this was not significant (Figure 14).

**Figure 15:**
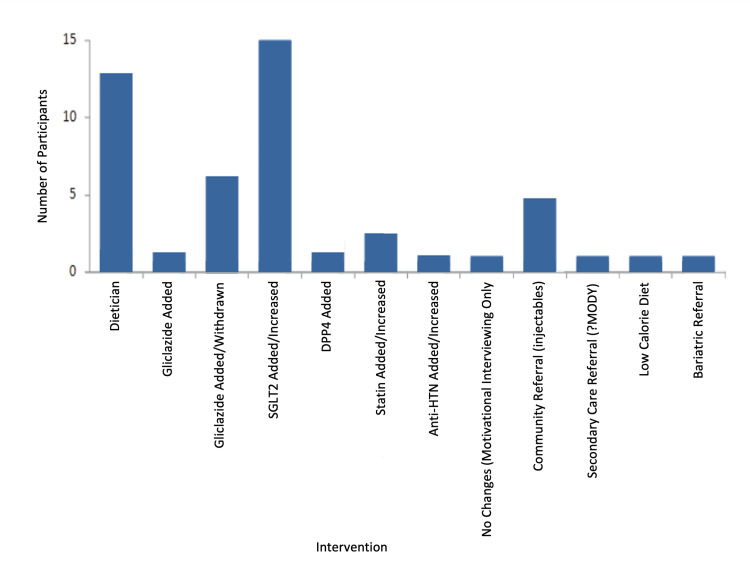
Intervention recorded after the initial optimisation clinic consultation. SGLT2: sodium-glucose co-transporter-2 inhibitor; HTN: hypertension; MODY: maturity-onset diabetes of the young

Of the 38 patients, 15 underwent medication adjustments, including introducing an SGLT2 inhibitor. Some started on empagliflozin 10 mg or canagliflozin 100 mg. Eight patients saw a gliclazide dosage reduction or discontinuation, while three had gliclazide added. Additionally, two patients began DPP4 inhibitor treatment, and three had increased statin dosages (Figure 15).

Thirteen patients received dietary consultations emphasising a reduction in carbohydrates, sugars, unhealthy fats, and red meat consumption. Five were referred for injectable therapy, one for a suspected maturity-onset diabetes of the young (monogenic) investigation, one for bariatric surgery, and another to the Low-Calorie Diet program in Waltham Forest.

**Figure 16:**
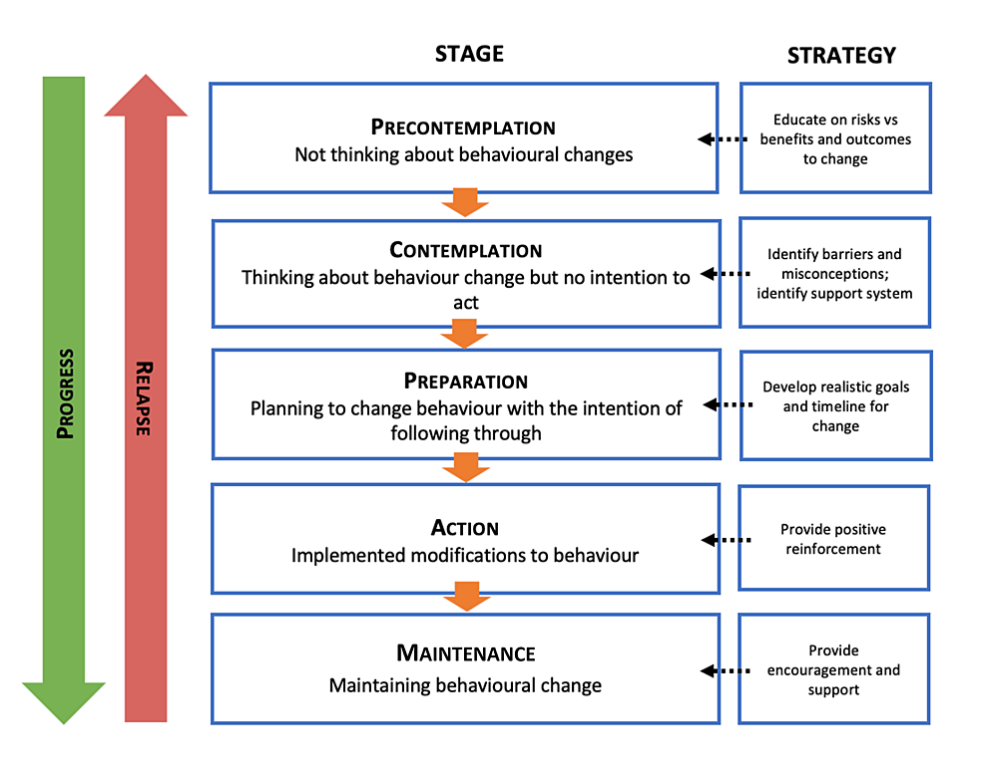
Theoretical model of behaviour change showing strategy used to encourage progress.

**Figure 17:**
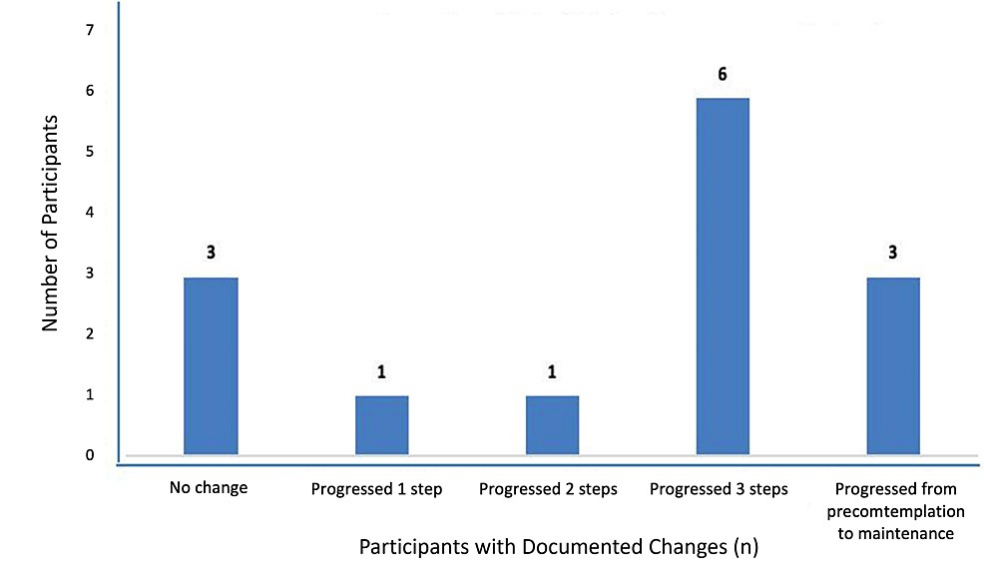
Study participants’ progress through the cycle of behaviour change.

Nine patients underwent tests for latent autoimmune diabetes in adults, all negative for markers such as glutamic acid decarboxylase, islet cell antibodies, islet antigen-2, and C-peptide. We documented and coded the participants’ behavioural changes, employing strategies specific to their stage. Of the 38 completing the pilot, 14 had behavioural stages recorded both initially and at follow-up. Eleven showed positive behavioural changes towards diabetes management (Figures 16, 17).

## Discussion

The recruitment goal for this pilot was 60 patients, and 43 were initially recruited. The goal was not met mainly due to the COVID-19 pandemic and the shift in patient priorities at the time. In addition, few participants were willing to engage as the pandemic prompted a switch to remote consultations. This meant that patients were contacted by telephone using a private number. Contacting participants over the telephone to describe the clinics’ objectives of optimising their care proved challenging despite our best efforts. Ultimately, the study recruited 38 (88%) participants who engaged fully. Engagement was assessed based on participants’ successful completion of initial and follow-up consultations and adherence to the necessary monitoring procedures. Those patients who met these criteria were included in the final statistical analysis. On the contrary, five (12%) patients initially recruited exhibited some level of participation but did not fulfil the engagement requirements and were, therefore, not included in the final statistical analysis.

Across all three participating practices, HbA1c levels significantly decreased, with the median HbA1c dropping from 88.0 mmol/mol to 70.0 mmol/mol (an average decrease from 93.6 to 69.4 mmol/mol). Triglyceride levels also saw a significant decline. While reductions in blood pressure, cholesterol ratio, total cholesterol, and BMI were not statistically significant, they trended towards lower values. Many patients benefited notably from behavioural interventions and motivational interviewing, enhancing their diabetes self-management, which bodes well for their long-term health.

This study highlighted the positive influence of behavioural change and motivational interviewing techniques on patients’ long-term diabetes self-management. Of the 38 patients, 15 needed medication adjustments, including the introduction of SGLT2 inhibitors. Interestingly, only 18% of patients were on a suitable individualised medication regimen before joining the optimisation clinic, pointing to the potential for enhanced primary care management.

Engaging patients proved challenging, especially for Practice 2, which had the most patients who did not engage compared to Practices 1 and 3. Significant administrative efforts focused on reaching these patients. However, among those who engaged, many showed improvements in diabetes self-management, highlighting the value of extended consultations and interview techniques.

Beyond glycaemic control, comprehensive diabetes management is vital, showcased by seminal studies like the United Kingdom Prospective Diabetes Study [6]. That study indicated that a reduction of HbA1c by 11 mmol/mol or more could result in a 37% drop in microvascular complications and overall mortality in T2D patients. Intensive glycaemic treatment can curtail both micro and macrovascular events [7]. Moreover, even achieving delayed glycaemic control in established diabetes can yield better outcomes for conditions such as retinopathy [8]. However, gradually improving glycaemic control is important, as rapid changes could heighten cardiovascular risk, especially in patients with longstanding diabetes [8]. No significant harm or severe hypoglycaemic episodes requiring hospitalisation were observed during our study. However, one patient experienced a drop in their HbA1c levels from 127 to 43 mmol/mol, which was attributed to their non-adherence to the medication regimen before participating in the pilot. This could theoretically accelerate retinopathy progression, underlining the importance of avoiding swift changes in glycaemic control [9].

SGLT2 inhibitors have revolutionised T2D management, decreasing all-cause mortality, cardiovascular events, and blood pressure [10]. Recent studies also highlight their therapeutic benefits in managing heart failure and renal disease [11,12]. With such advancements in OHAs, the establishment of optimisation clinics offers physicians a valuable avenue for transitioning diabetic patients from older, less effective medications to newer, more effective alternatives, often in conjunction with lifestyle interventions. Additionally, the availability of newer OHAs, including SGLT2 inhibitors, expands the possibilities for fine-tuning treatment objectives without the need for injectable therapies such as insulin.

Study participants showed significant improvements in metabolic profiles, especially reductions in HbA1c and triglycerides. Most achieved their target blood pressure and lipid levels, indicating that primary care practitioners effectively managed these conditions. This efficiency hints at the potential for achieving other treatment targets such as HbA1c. Although many patients were not fully optimised on OHAs, only five of the original 43 (12%) were considered for injectable therapy. This suggests further potential for OHA optimisation before pilot enrolment.

With appropriate training, future optimisation clinics could introduce glucagon-like peptide-1 agonists or basal insulin therapy in primary care settings. Prior research has demonstrated that a substantial decrease in HbA1c levels and improvements in metabolic parameters, such as the results from our study, correlate with a decreased risk of vascular disease [13]. Considering the growing economic impact of T2D, the optimisation pilot offers a promising strategy for enhancing diabetes management and patient empowerment.

In 2010, the United Kingdom’s estimated costs for T2D were approximately £21.8 billion. Projections suggest that by 2035, this cost will rise to £36 billion [14]. However, this pilot project’s total cost was £10,000, benefitting 38 patients, a modest expense of around £263 per patient.

While the benefits of tight glycaemic control are well-known, there is room to further investigate diabetes optimisation services at the PCN level in London. The Tier 2 Diabetes Support Team was introduced to enhance primary care diabetes management through a multidisciplinary team, including a general practitioner (GP) with an extended diabetes role, a practice nurse, clinical pharmacists, and community diabetes specialists. Unfortunately, the London Borough of Barnet had to discontinue the service after two years due to funding challenges, despite its positive impact on patient care. This emphasised the need for improved integration between primary and secondary healthcare pathways to enhance diabetes care [15].

Our study provides insights into primary care-led services requiring a lead GP and a dedicated administrator. Our study met its objectives and proved cost-effective on a per-patient basis, requiring a smaller workforce. Additionally, it demonstrated the rapid establishment of remote clinics, even during a pandemic, ensuring uninterrupted diabetes care in resource-constrained areas. While primary care-led clinics are not novel, this approach is innovative due to its cost-effectiveness, emphasis on behavioural modifications, and proactive response to a challenging healthcare issue with limited existing solutions.

Interestingly, this study revealed that participants could be categorised into three groups: Group A consisted of individuals actively managing their diabetes; Group B were individuals initially hesitant but experiencing significant physical and mental improvements after active recall and engagement; and Group C consisted of individuals not currently motivated to manage their diabetes for whom engagement efforts required substantial resources and yielded limited success. The study emphasised the importance of extended consultations and periodic specialist reviews, especially for those in Groups A or B.

Recommendations

Our pilot study highlights the potential of primary-care-led optimisation clinics in addressing the challenges of T2D. A dedicated administrator proved invaluable, permitting doctors to dedicate their entire focus to patient consultations. Furthermore, a collaborative effort with secondary care was particularly fruitful. This partnership facilitated extensive patient coverage through virtual reviews, enriching the understanding within primary care. More importantly, the collaboration showcased the significance of timely optimisation of oral therapy, which promises to reduce complications and referrals.

Considering the broader picture, introducing a robust recall system and setting up specialised optimisation clinics appear imperative. Such measures promise consistent patient engagement and offer a cost-effective solution. When juxtaposed with the long-term expenses of diabetes complications, the administrative costs of these clinics seem warranted.

Navigating the complexities of T2D patient care mandates that healthcare providers maintain regular patient contact. Notably, the pilot’s findings, which evidenced promising improvements in HbA1c levels and triglyceride concentrations, should be seen within the larger framework of diabetes management. However, given the observed declining trends in other metabolic parameters, there is a pressing need for longer studies. Such extended research will more conclusively determine the enduring efficacy of the interventions applied.

Limitations

The pilot was conducted at the height of the COVID-19 pandemic. Individuals with diabetes, as evidenced, bore an elevated risk from COVID-19, further exacerbating their diabetes management [16]. Previous studies corroborate that glycaemic control in T2D patients suffered during this period, irrespective of national lockdown protocols [17,18]. These adverse outcomes, compounded by the diverse psychosocial stressors of the pandemic, provide a plausible explanation for the decreased engagement levels detected in our pilot.

Another important limitation of the study was the restricted sample size and the overarching influence of the COVID-19 pandemic. The latter might have reoriented patients’ approach towards diabetes management. Such a shift could have led to decreased adherence to prescribed treatments or behavioural changes, reflected in the data as reduced patient engagement. Moreover, the enforced reliance on remote consultations, a direct consequence of pandemic-related social distancing norms, may have inadvertently diminished the true impact of the interventions. Consequently, future research would benefit from integrating face-to-face consultations, ensuring a richer, more personalised patient experience.

The financial limitations of the NHS also pose considerable challenges. A larger-scale study would necessitate securing funds for these clinics. Such an endeavour would involve recruiting or reallocating dedicated administrators and primary care physicians within each PCN. This would ensure the catering to the needs of T2D patients, especially when primary care faces heightened demand.

## Conclusions

PCN-based optimisation clinics can markedly improve T2D management within the NHS, reducing referrals. Although more research is needed, our study points to the potential for patient optimisation at the primary care level. The evident benefits of optimisation clinics include in-depth patient discussions about diabetes management challenges. Collaborating with an endocrinologist offered expert management and guidance, alleviating the referral burden on secondary care, thus establishing it as a cost-effective solution. As our understanding of diabetes evolves alongside pharmaceutical advancements, it remains imperative to prioritise motivational interviewing and patient empowerment in diabetes care.
